# *TGIF1* and *SF1* polymorphisms are associated with litter size in Small Tail Han sheep

**DOI:** 10.1111/rda.13753

**Published:** 2020-07-07

**Authors:** Zhuangbiao Zhang, Xiaoyun He, Qiuyue Liu, Jishun Tang, Ran Di, Mingxing Chu

**Affiliations:** ^1^ Key Laboratory of Animal Genetics, Breeding and Reproduction of Ministry of Agriculture and Rural Affairs Institute of Animal Science Chinese Academy of Agricultural Sciences Beijing China; ^2^ Institute of Animal Husbandry and Veterinary Medicine Anhui Academy of Agricultural Sciences Hefei China

**Keywords:** litter size, *SF1*, sheep, SNPs, *TGIF1*

## Abstract

*TGF‐β induced factor homeobox 1* (*TGIF1*) and *splicing factor 1* (*SF1*) are important for mammalian reproduction; however, the effects of these genes on litter size in sheep remain unexplored. In this study, we genotyped 768 ewes from seven sheep breeds at two loci: g.37871539C>T, a synonymous mutation of *TGIF1*; and g.42314637T>C, a 3′UTR variant of *SF1*. Our analysis of polymorphism revealed only two genotypes at locus g.37871539C>T in *TGIF1*, with most sheep populations being moderately polymorphic (0.25 < PIC < 0.5) at this site. In contrast, most breeds exhibited low polymorphism (PIC ≤0.25) at the *SF1* locus g.42314637T>C. The association analysis revealed that a synonymous mutation at g.37871539C>T in *TGIF1* was highly associated with litter size in Small Tail Han sheep, in which it causes a significant decrease in litter size. Conversely, while the *SF1* 3′UTR variant g.42314637T>C was also highly associated with litter size in sheep, it causes a significant increase in the number of litter size. Combined, these data provide valuable information regarding candidate genetic markers for sheep breeding programs.

## 1 INTRODUCTION

Understanding the genetics of reproduction is important for the sheep industry, and recent researches have revealed that the regulation of litter size in sheep and goats is multiallelic and polygenic (Wang et al., 2019; Zhang et al., 2020). For example, *FecB* is a prominent gene affecting litter size, and ewes with the genotype of FecB BB carry two copies of a FecB mutation. This increases their ovulation rate and litter size by 3 and 1.5, respectively, over ewes with the genotype of FecB++. Likewise, ewes with one copy of the FecB mutation (FecB B+) have an ovulation rate and litter size 1.5 and 1 greater than FecB++ ewes, respectively (Liu et al., 2014). Mutations in a number of other genes have also been demonstrated to be useful genetic markers for sheep breeding. These genes include *bone morphogenetic protein 15* (*BMP15*; Calvo et al., 2020), *growth differentiation factor 9* (*GDF9*; Våge, Husdal, Kent, Klemetsdal, & Boman, 2013), *melatonin receptor 1A* (*MTNR1A*; He, Zhang, Liu, & Chu, 2019), *BMP7* and *BMP2* (Zhang, Liu, et al., 2019). Such findings indicate that the identification of genetic markers can facilitate the progress of molecular marker‐assisted breeding in sheep.

TGF‐β induced factor homeobox 1 (TGIF1) is a member of the three‐amino‐acid loop‐extension (TALE) superfamily that is highly expressed in human (Hu, Yu, Shaw, Renfree, & Pask, 2011) and sheep (Jiang et al., 2014) ovaries, and highly conserved in mammals (Shen & Walsh, 2005). This suggests that TGIF1 plays a critical role in mammalian reproduction. Studies have also revealed that TGF‐β/SMAD signalling is important in reproductive processes, including in follicular activation (Yin, Chang, Yi, Yao, & Leung, 2020), ovarian follicle development (Knight & Glister, 2006) and oocyte maturation (Yin et al., 2020). Indeed, the inhibition or mutation of key members of the TGF‐β/SMAD signalling pathway can cause reproductive problems (Chand, Robertson, Shelling, & Harrison, 2007), including sterility (Li et al., 2017). Significantly, TGIF1 in human and zebra fish functions as a repressor, and reversibly modulates important members of the TGF‐β/SMAD signalling pathways that are activated by TGF‐β, such as *SMAD2* and *SMAD4* (Hu et al., 2011; Hyman, Bartholin, Newfeld, & Wotton, 2003; Wotton, Lo, Lee, & Massagué, 1999). Therefore, TGIF1 may be a crucial factor in the reproduction of sheep and other mammalian species.

Mammalian splicing factor 1 (SF1) is indispensable for recognizing pre‐mRNA 3′ splice sites during the process of early spliceosome assembly (Selenko et al., 2003). SF1 also participates in the formation of extraspliceosomal complexes (Rino, Desterro, Pacheco, Gadella, & Carmo‐Fonseca, 2008), which may influence the expression of other genes. Further studies have suggested that SF1 plays important functions in alternative splicing (Corioni, Antih, Tanackovic, Zavolan, & Krämer, 2010), and notably, alternative splicing events in genes such as *oestrogen receptor alpha* (Schoen, Sharbati, Ritter, & Jewgenow, 2013) and *kisspeptin‐1 receptor* (Mechaly, Viñas, & Piferrer, 2009) were involved in reproduction. Also, Miao, Luo, Zhao, and Qin (2018) revealed that a number of alternative splicing events are associated with ewe fecundity in diverse sheep breeds, by analysing ovarian transcriptomes. Together, these findings suggest that SF1 may be a factor affecting sheep reproduction.

In this study, we examined the association between *TGIF1* and *SF1* polymorphisms and litter size in Small Tail Han sheep. This is the first analysis of such data, and our results provide useful genetic markers for future sheep breeding programs.

## 2 MATERIALS AND METHODS

### 2.1 Animal and sample preparation

All experimental procedures involving animals were approved by the Science Research Department (in charge of animal welfare issues) of the Institute of Animal Science, Chinese Academy of Agricultural Sciences (IAS‐CAAS; Beijing, P. R. China). Ethics approval was granted by the animal ethics committee of IAS‐CAAS (No. IASCAAS‐AE‐03; 12 December 2016).

This study began in June, 2016. Total DNA was extracted from blood samples obtained from the jugular veins of 768 ewes representing seven sheep breeds. The higher prolificacy breeds included in this study were the Small Tail Han sheep (*n* = 384), Hu sheep (*n* = 83) and Cele Black sheep (*n* = 68). The remaining four breeds were the Sunite sheep (*n* = 70), Prairie Tibetan (*n* = 80) and Suffolk sheep (*n* = 60) and Tan sheep (*n* = 23); these are lower prolificacy breeds (Table 1). All ewes used in this study were randomly selected and had been fed in the same regions since they were born, with no sire effects. All sheep were freely mated within their breeds.

**TABLE 1:** The numbers and sources of ewes used in this study

Breed	Number	Type	District
Small Tail Han sheep	384	Polytocous type	Southwest Region, Shandong Province, China
Hu sheep	83	Polytocous type	Xuzhou, Jiangsu Province, China
Cele Black sheep	68	Polytocous type	Cele, Xinjiang Uygur Autonomous Region, China
Sunite sheep	70	Monotocous type	Wulatezhongqi, Bayannaoer, Inner Mongolia Autonomous Region, China
Prairie Tibetan sheep	80	Monotocous type	Dangxiong, Tibet Autonomous Region, China
Suffolk sheep	60	Monotocous type	Beijing Aoxin Stud Farm Co. Ltd. located in Shunyi District, Beijing, China
Tan sheep	23	Monotocous type	Yanchi, Ningxia Hui Autonomous Region, China

### 2.2 Primer design and genotyping

Primers to genotype the loci g.37871539C>T of *TGIF1* and g.42314637T>C of *SF1* were designed by MassARRAY Assay Design v.3.1, according to sequences of *TGIF1* and *SF1* deposited in GenBank (accession numbers: NC_019480.1, *TGIF1*; NC_019478.1, *SF1*). These primers were synthesized by Beijing Compass Biotechnology Co., Ltd., and the genotyping process was conducted using the MassARRAY^®^ system. The MassARRAY is a recently developed strategy for the detection of single nucleotide polymorphisms (SNPs). Initially, the amplification of target sequences is conducted (primers are shown in Table 2); subsequently, single‐base extended PCR reactions are performed using specific primers (Table 2). The molecular weight of the amplified products differs after extension because different bases are present at polymorphic sites. These differences are then detected through time‐of‐flight mass spectrometry, which allows the products to be separated based on genotype. The MassARRAY^®^ SNP detection system has been described in further detail elsewhere (He et al., 2019; La, Liu, Zhang, & Chu, 2019; Zhang, Liu, et al., 2019; Zhou et al., 2018).

**TABLE 2:** Primer information for genotyping

Primer	Sequence	Product size	Usage
TGIF1‐F	ACGTTGGATGATACAGCAGATGGCCAACAG	94	PCR amplification
TGIF1‐R	ACGTTGGATGGGTCCAGATTTACAGGAGTC		
TGIF1‐E	ACAGCAGCTTTACAGA		single‐base extended PCR amplification for g.37871539C>T
SF1‐F	ACGTTGGATGGGGTCTCTCATTCCTCTCAG	92	PCR amplification
SF1‐R	ACGTTGGATGGTGAGTGGAGTATTTTGGGC		
SF1‐E	GCCCCACCCTCCCAA		single‐base extended PCR amplification for g.42314637T>C

### 2.3 Statistical analyses

Based on genotyping data, we calculated allele and genotype frequencies, polymorphism information content (PIC), heterozygosity (HE), and the number of effective alleles (NE) and *p* values for chi‐square tests. If a particular ewe population had *p* > .05 (chi‐square test), it was considered to be under Hardy–Weinberg equilibrium. Regarding an association between *TGIF1* and *SF1* polymorphisms and litter size, chi‐square test was performed to analyse the association of litter size with genotypes in each parity of Small Tail Han sheep.

### 2.4 Prediction of protein interaction networks via the STRING database

As proteins modulate most physiological activities, identifying the interactions between proteins can enhance our understanding of complex traits such as litter size. To investigate the mechanisms by which TGIF1 and SF1 may modulate ovulation and litter size, we predicted the protein interaction networks of TGIF1 and SF1 using the STRING database (https://string‐db.org).

## 3 RESULTS

### 3.1 Genotyping and population genetic analysis of g.37871539C>T in *TGIF1* and g.42314637T>C in *SF1 * in seven sheep breeds

**FIGURE 1:**
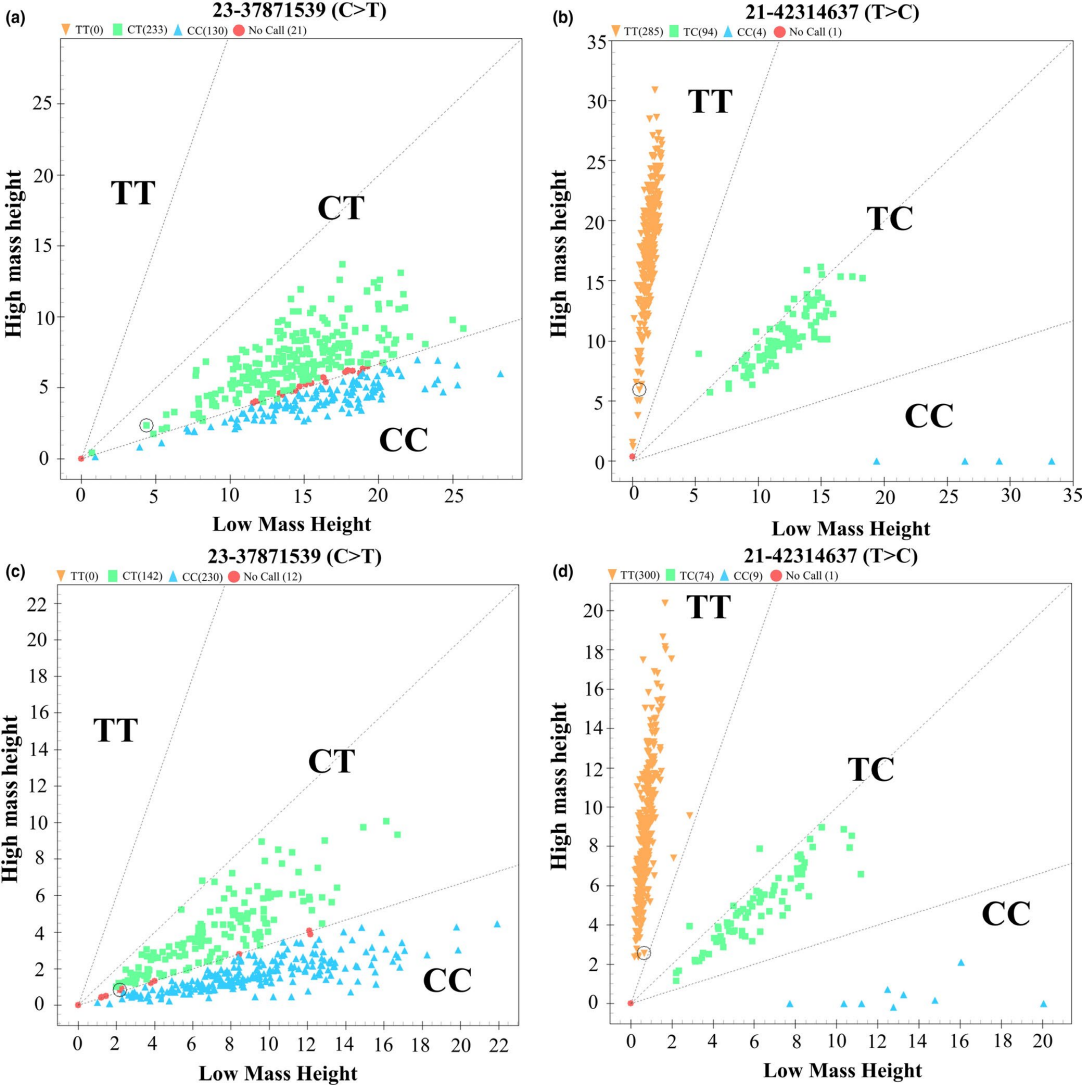
Genotyping of SNPs at reproduction‐related loci in sheep. The genotyping results of g.37871539C>T in *TGIF1* (a) and g.42314637T>C in *SF1* (b) in six sheep breeds (Hu sheep, Cele Black sheep, Sunite sheep, Prairie Tibetan sheep, Suffolk sheep and Tan sheep). The genotyping results of g.37871539C>T in *TGIF1* (c) and g.42314637T>C in *SF1* (d), in Small Tail Han sheep

The genotyping results were showed in (Figure 1). The results suggested that only two genotypes were detected at locus g.37871539C>T (*TGIF1*), while three genotypes were detected at locus g.42314637T>C (*SF1*. Our population genetic analysis (Table 3) showed that the locus g.37871539C>T in *TGIF1* (synonymous mutation) contained only two genotypes across the seven sheep breeds studied. Most of these breeds exhibited moderate polymorphism at this site (0.25 < PIC < 0.5), while the Suffolk sheep exhibited relatively low polymorphism. Only two breeds (Suffolk sheep and Tan sheep) were under Hardy–Weinberg equilibrium at this locus (*p* > .05). Regarding locus g.42314637T>C in *SF1* (3′UTR variant), three sheep breeds (Small Tail Han sheep, Cele Black sheep and Suffolk sheep) possessed three genotypes; the remaining four breeds had two genotypes only. The Cele Black sheep was moderately polymorphic for this locus (0.25 < PIC < 0.5), while the other six breeds exhibited lower polymorphism. With the exception of the Prairie Tibetan sheep, this locus was under Hardy–Weinberg equilibrium in all breeds (*p* > .05).

**TABLE 3:** Population genetic analysis of g.37871539C>T (*TGIF1*) and g.42314637T>C (*SF1*) in seven sheep breeds

Locus (mutation type)	Breed	Genotype frequency			Allele frequency		PIC	HE	NE	Chi‐square test (*p* value)
g.37871539C>T (synonymous mutation)		CC	CT	TT	C	T				
	Small Tail Han Sheep	0.57	0.43	0.00	0.79	0.21	0.28	0.34	1.51	.00
	Hu sheep	0.26	0.74	0.00	0.63	0.37	0.36	0.47	1.87	.00
	Cele Black sheep	0.16	0.84	0.00	0.58	0.42	0.37	0.49	1.95	.00
	Sunite sheep	0.39	0.61	0.00	0.69	0.31	0.33	0.42	1.74	.00
	Prairie Tibetan sheep	0.22	0.78	0.00	0.61	0.39	0.36	0.48	1.91	.00
	Suffolk sheep	0.79	0.21	0.00	0.90	0.10	0.17	0.19	1.23	.38
	Tan sheep	0.55	0.45	0.00	0.78	0.22	0.29	0.35	1.54	.19
g.42314637T>C (3′UTR variant)		TT	TC	CC	T	C				
	Small Tail Han Sheep	0.78	0.19	0.03	0.88	0.12	0.19	0.21	1.27	.09
	Hu sheep	0.94	0.06	0.00	0.97	0.03	0.06	0.06	1.06	.78
	Cele Black sheep	0.57	0.38	0.05	0.76	0.24	0.30	0.36	1.56	.61
	Sunite sheep	0.74	0.26	0.00	0.87	0.13	0.20	0.22	1.29	.22
	Prairie Tibetan sheep	0.64	0.36	0.00	0.82	0.18	0.25	0.30	1.42	.05
	Suffolk sheep	0.75	0.23	0.02	0.87	0.13	0.20	0.23	1.30	.94
	Tan sheep	0.91	0.09	0.00	0.96	0.04	0.08	0.08	1.09	.83

### 3.2 The association of *TGIF1* and *SF1* polymorphisms with litter size in Small Tail Han sheep

Across three parities, the litter size of ewes with the wild‐type genotype CC at locus g.37871539C>T (*TGIF1*) was higher than that of ewes with the genotype CT (*p* < .05; Table 4), this result means this mutation causes a significant decrease in litter size. Regarding locus g.42314637T>C (*SF1*), the litter size of ewes with the mutant homozygous genotype CC, over three parities, was higher than that of ewes with the genotype TT (*p* < .05), this result means this mutation causes a significant increase in litter size.

**TABLE 4:** The mean, standard deviations (*SD*) and *p* value (chi‐squared test) of litter size in Small Tail Han sheep in the loci g.37871539C>T (*TGIF1*) and g.42314637T>C (*SF1*)

Locus	Genotype	Litter size (mean ± *SD*)					
		First parity (*N*)	*p* value	Second parity (*N*)	*p* value	Third parity (*N*)	*p* value
g.37871539C>T	CC	2.12 (230) ± 0.688	.045	2.32 (121) ± 0.698	.024	2.67 (51) ± 0.817	.001
	CT	1.80 (142) ± 0.618		1.99 (84) ± 0.703		2.11 (38) ± 0.727	
g.42314637T>C	TT	1.86 (300) ± 0.648	.035	2.10 (186) ± 0.754	.042	2.35 (66) ± 0.816	.038
	TC	1.94 (74) ± 0.703		2.28 (45) ± 0.815		2.59 (17) ± 0.944	
	CC	2.43 (9) ± 0.707		2.63 (9) ± 0.527		3.00 (8) ± 0.000	

Note

*p* < .05 in each column indicates a significant difference.

### 3.3 Protein interaction networks involving TGIF1 and SF1

To investigate the mechanisms by which TGIF1 and SF1 might affect ovulation and litter size in sheep, we predicted protein interaction networks involving TGIF1 and SF1 via the STRING database. This analysis suggested that both TGIF1 and SF1 interact with ten other proteins (Figure S1).

## 4 DISCUSSION

### 4.1 *TGIF1* and *SF1* polymorphism in sheep

*TGIF1* and *SF1* may be important regulators of reproduction (Corioni et al., 2010; Hu et al., 2011; Hyman et al., 2003; Schoen et al., 2013; Wotton et al., 1999); however, the extent and effects of their polymorphisms in sheep are poorly understood. At the loci studied here, we detected only two *TGIF1* genotypes in all ewe populations and two *SF1* genotypes in some ewe populations (Table 3); some of these populations also exhibited low polymorphism (PIC≤0.25). These data may be limited by the number of sheep studied, and thus increasing the sample size could enable three genotypes to be captured, which would also increase the PIC value. Importantly, considering the negative effects of mutation on litter size, the lethality is the most likely reason for lack of TT genotype (g.37871539C>T) in all sheep breeds. Furthermore, we found that several sheep breeds were not in Hardy–Weinberg equilibrium (*p* ≤ .05) at the studied loci (Table 3), which may be a consequence of selection.

### 4.2 Analysis of the association between *TGIF1 *and *SF1* polymorphisms and litter size

Missense mutations cause the substitution of one amino acid for another and can therefore exert great influence on complex traits. For example, point mutations in *BMP15* (Calvo et al., 2020), *GDF9* (Våge et al., 2013), *MTNR1A* (He et al., 2019), *BMP7* and *BMP2* (Zhang, Liu, et al., 2019) significantly affect litter size in sheep. Recently, synonymous mutations have also received attention for playing key roles in reproductive traits. For example, a synonymous mutation of *melatonin receptor 1A* was found in Rasa Aragonesa sheep and was highly associated with reproductive seasonality (Martínez‐Royo, Lahoz, Alabart, Folch, & Calvo, 2012); a synonymous mutation of *luteinizing hormone beta polypeptide* was detected in Small Tail Han sheep and was highly associated with litter size (Wang et al., 2020); and a synonymous mutation in *follicle‐stimulating hormone receptor* was highly associated with litter size in sheep, including in Small Tail Han and Hu sheep (Pan et al., 2014). These findings indicate that synonymous mutations have the potential to be valuable markers for improving sheep fecundity.

TGIF1 and SF1 act as regulators in reproductive processes (Corioni et al., 2010; Hu et al., 2011; Hyman et al., 2003; Schoen et al., 2013; Wotton et al., 1999); however, their effects on litter size remain to be explored. Here, we conducted an association analysis of *TGIF1* and *SF1* polymorphisms with litter size in Small Tail Han sheep. Regarding locus g.37871539C>T in *TGIF1*, only two genotypes were identified, and litter size in ewes with the genotype CT was significantly lower than that of ewes with the wild‐type genotype CC. This suggests that this synonymous mutation may be harmful. Furthermore, we failed to detect the mutant homozygous genotype TT, and the lethality was the most likely reason for lack of TT genotype (g.37871539C>T) in all sheep breeds. TGIF1 can negatively regulate TGF‐β‐activated genes such as *SMAD2* and *SMAD4* (Hu et al., 2011; Hyman et al., 2003; Wotton et al., 1999), and TGF‐β/SMAD signalling plays crucial roles in mediating reproduction (Knight & Glister, 2006; Liu, Chang, Yi, Yao, & Leung, 2019; Rossetti et al., 2020; Yin et al., 2020). For example, the disruption of TGF‐β/SMAD signalling can cause reproductive problems (Chand et al., 2007), including sterility (Li et al., 2017). Therefore, while on one hand, our failure to detect the third genotype at the g.37871539C>T locus may be due to inadequate ewe numbers; on the other hand, we hypothesize that this synonymous mutation may enhance the negative regulatory effects of TGIF1 on TGF‐β/SMAD signalling, and even cause death in embryos with the homozygous TT genotype.

*SF1* is mainly involved in alternative splicing events (Corioni et al., 2010), thus polymorphism in the 3′UTR of *SF1* could modulate gene stability (Akdeli et al., 2014) and influence protein abundance. Considering the key roles reported for alternative splicing in sheep reproduction (Miao et al., 2018), we speculate that the *SF1* 3′UTR mutation g.42314637T>C may increase litter size by regulating alternative splicing events and modifying protein abundance related to reproduction.

### 4.3 Proteins interacting with TGIF1 and SF1

Most physiological processes are underpinned by multiple proteins; therefore, building interaction networks of proteins can enhance our understanding of complex traits such as reproduction. In our analysis, TGIF1 was predicted to interact with SMAD2, which has been reported to participate in reproductive processes. Specifically, several studies have suggested that SMAD2 may be a candidate gene influencing reproduction in sheep with different fecundity (Zhang, Tang, et al., 2019; Zheng et al., 2019), and the inactivation of SMAD2 in mouse can lead to endometrial dysregulation and infertility (Kriseman et al., 2019). Previous study highlighted that the TGF‐β induced factor homeobox 2 (TGIF2) has the same DNA binding homeodomains as TGIF1, suggesting that these two proteins may bind the same regulatory sequences (Melhuish, Gallo, & Wotton, 2001). As TGIF2 has been detected in tammar granulosa and theca cells, indicating a role in folliculogenesis (Hu et al., 2011), we speculate that TGIF1 may also be involved in this process.

SF1 was predicted to interact with ten proteins, including cell division cycle 5‐like protein (CDC5L) and U2 small nuclear RNA auxiliary factor 2 (U2AF2). While CDC5L is a key factor regulating the cell cycle (Zhang, Kaur, Akhter, & Legerski, 2009), it is also highly abundant in BCB‐positive GV oocytes (Liu et al., 2018) and follicles larger than eight mm‐including prematuration oocytes (Dieleman et al., 2002). CDC5L could therefore act as a regulator promoting oocyte development and maturation. U2AF2 plays an important role in splicing decisions and has been reported to participate in alternative splicing events (Sutandy et al., 2018), such events have also been associated with reproduction (Miao et al., 2018).

## 5 CONCLUSIONS

This study reported the first analysis of polymorphisms at two loci in *TGIF1* and *SF1*, and suggested that these loci (g.37871539C>T in *TGIF1* and g.42314637T>C in *SF1*) were highly associated with litter size in sheep. All in all, these data provide valuable genetic markers for sheep breeding.

## CONFLICT OF INTEREST

None of the authors have any conflict of interest to declare.

## AUTHOR CONTRIBUTIONS

Zhuangbiao Zhang and Mingxing Chu designed this study; Xiaoyun He, Qiuyue Liu and Jishun Tang collected the data used in this study; Zhuangbiao Zhang, Xiaoyun He and Ran Di performed statistical analysis; Zhuangbiao Zhang drafted paper; Mingxing Chu revised this manuscript.

## Supporting information

Fig S1

## Data Availability

Data sharing is not applicable to this article as no new data were created or analysed in this study.
