# Supplementary figures and images for: TGIF1 and SF1 polymorphisms are associated with litter size in Small Tail Han sheep
Source: Reprod Domest Anim. 2020 Jul 7;55(9):1145–53. doi: 10.1111/rda.13753 (PMC7540012; doi:10.1111/rda.13753)

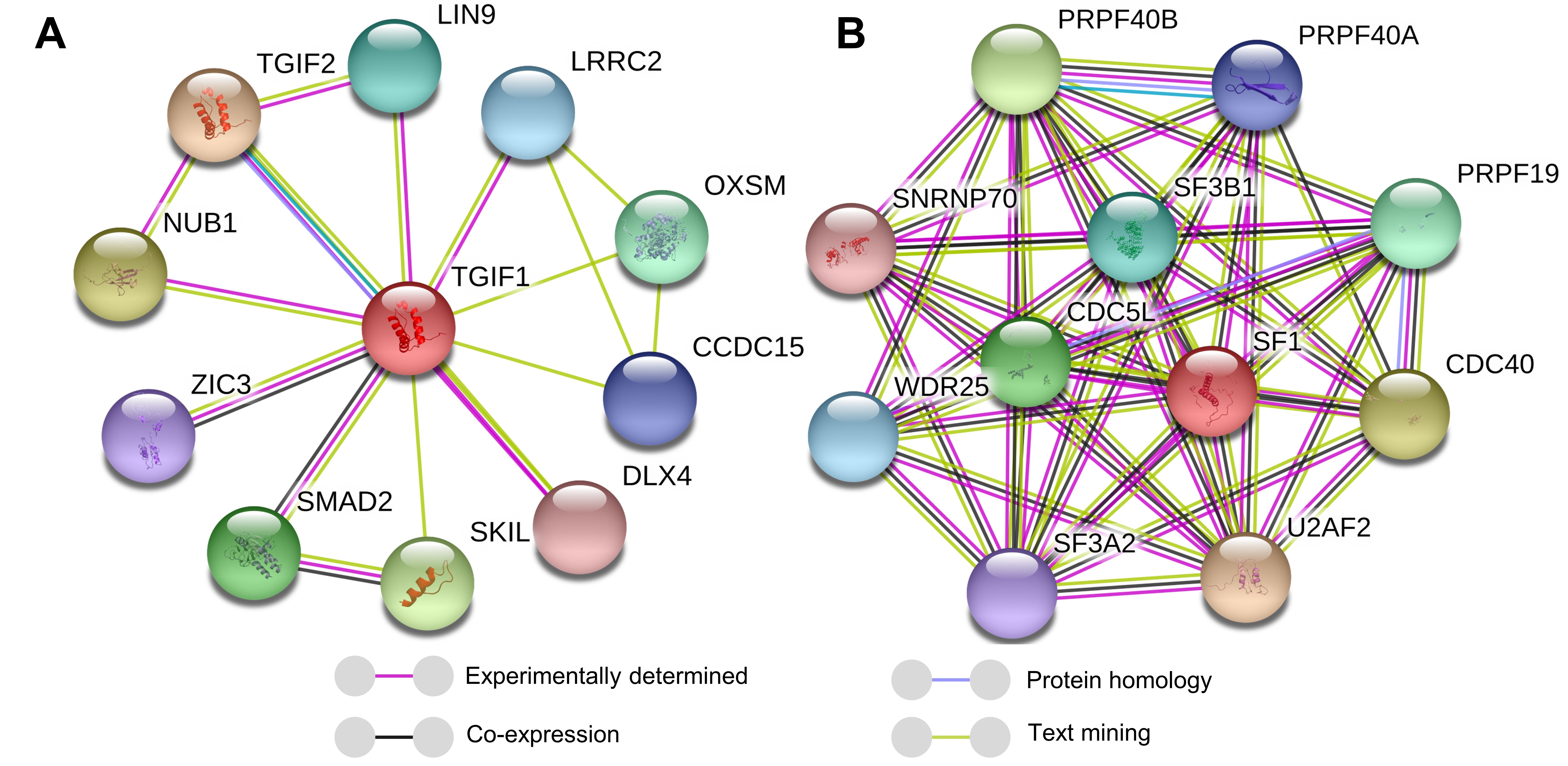

Supplement: Supplementary file 1 — Fig S1 [file RDA-55-1145-s001.jpg]
